# Underwater precutting endoscopic mucosal resection using a multifunctional snare for a large colonic laterally spreading tumor

**DOI:** 10.1055/a-2072-3383

**Published:** 2023-05-10

**Authors:** Kazuo Shiotsuki, Yorinobu Sumida, Mitsuru Esaki, Yosuke Minoda, Shin-ichiro Fukuda, Eikichi Ihara, Hirotada Akiho

**Affiliations:** 1 Department of Gastroenterology, Kitakyushu Municipal Medical Center, Kitakyushu, Fukuoka, Japan; 2 Department of Medicine and Bioregulatory Sciences, Graduate School of Medical Sciences, Kyushu University, Fukuoka, Fukuoka, Japan; 3 Department of Gastroenterology and Metabolism, Graduate School of Medical Sciences, Kyushu University, Fukuoka, Fukuoka, Japan

Underwater endoscopic mucosal resection (UEMR) is a recently developed procedure characterized by resection of the colon polyps immersed in water. Thanks to the “floating effect” on the mucosa and submucosa, UEMR allows snaring the lesion much more easily compared to conventional EMR 1 2 . However, it is technically challenging to use UEMR for large colon polyps, especially a large colonic laterally spreading tumor (LST), due to the risk of incomplete resection. In contrast, precutting EMR (PEMR) with a planned partial circumferential mucosal incision is reportedly useful for en bloc resection of a colonic LST 3 . We considered that UEMR would complement PEMR, exerting synergistic effects. Here we present a case of a large colonic LST successfully treated by UEMR in conjunction with underwater PEMR ( Video 1 ).

**Video 1** Underwater precutting endoscopic mucosal resection using a multifunctional snare for a large laterally spreading tumor.


Colonoscopy revealed a flat elevated polyp 30 mm in size extending along a semilunar fold in the descending colon. Magnifying endoscopy was indicative of adenoma ( Fig. 1 ). Underwater PEMR using a multifunctional device equipped with both a snare and knob-shaped tip was applied to this lesion 4 5 ( Fig. 2 ). At the proximal side of the lesion in immersed water, a partial mucosal incision was made with the tip of the snare following local injection of hyaluronic acid solution ( Fig. 3 ). By fixing the tip of the snare to the proximal mucosal incision, the lesion was easily introduced into the snare under a condition of immersed water. En bloc resection was achieved without any complications ( Fig. 4 ). The total procedure time was only 11 minutes. Histopathology revealed an adenoma with negative margins ( Fig. 5 ).

**Fig. 1:**
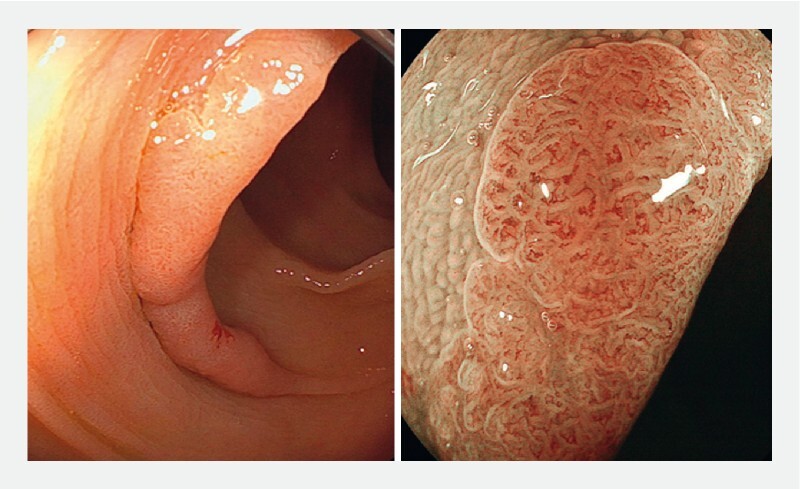
Colonoscopy showed a large flat elevated polyp extending along a semilunar fold in the descending colon. Blue laser imaging magnifying endoscopy was indicative of an adenoma.

**Fig. 2:**
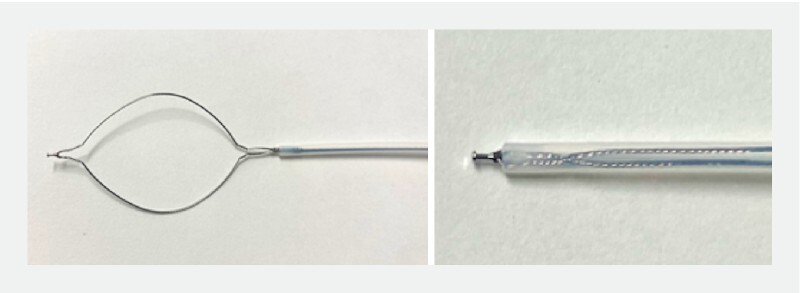
A picture of a multifunctional snare equipped with both the snare and knob-shaped tip used in this case. A knob-shaped tip is attached to the top of the snare.

**Fig. 3:**
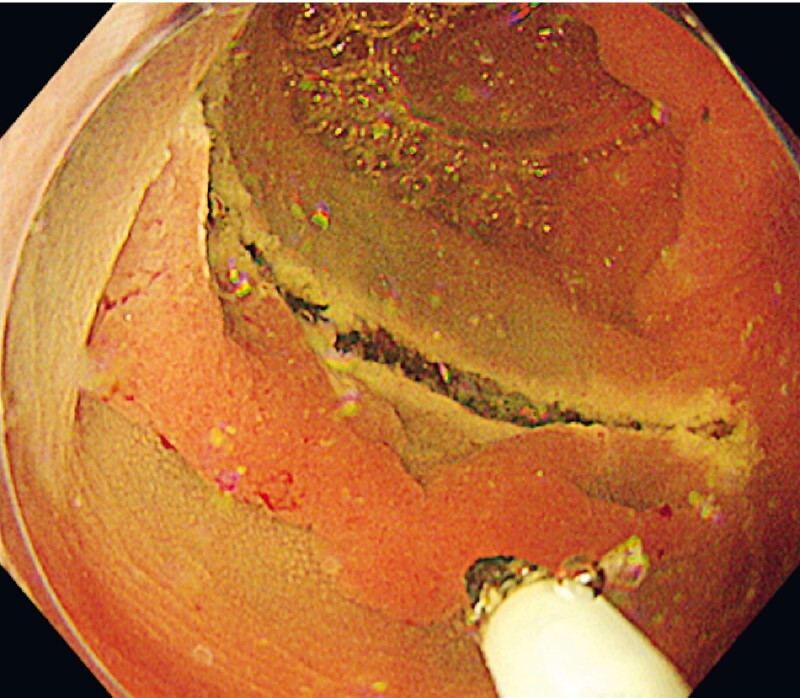
Planned mucosal incision was performed at the proximal side of the lesion using the tip of a multifunctional snare.

**Fig. 4:**
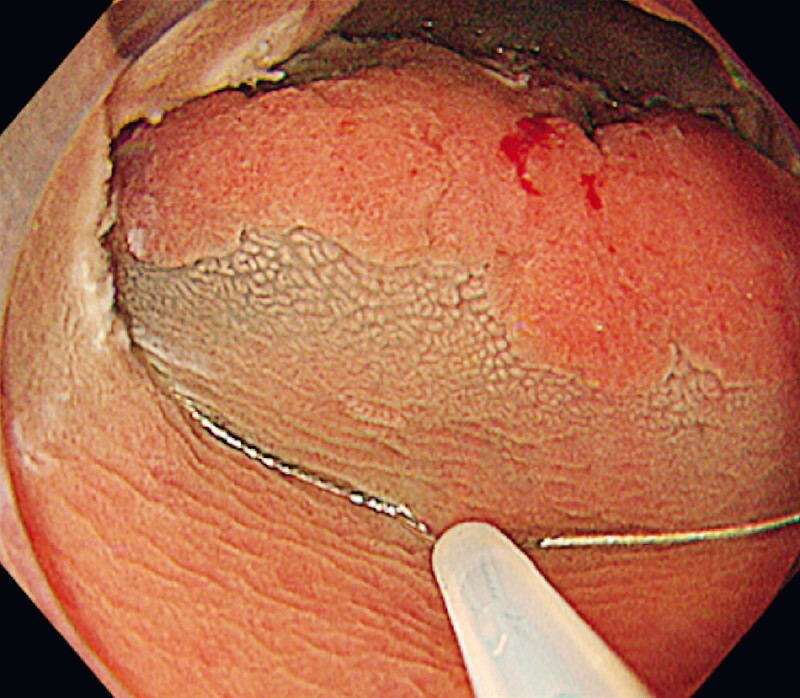
After fixing the tip of the snare to the proximal mucosal incision, the lesion was introduced into the snare under a condition of immersed water.

**Fig. 5:**
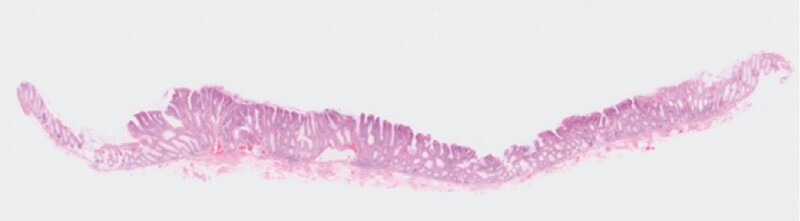
Histopathological findings of the laterally spreading tumor successfully treated by underwater precutting endoscopic mucosal resection. A low magnification view showed an adenoma with negative resection margins.

The present case has shown that UEMR exerts synergic effects with PEMR for a large colonic LST. Another important advantage of underwater PEMR is that it can be completed with a single device. Underwater PEMR using a multifunctional device could be a good option for treatment of a large colonic LST.

Endoscopy_UCTN_Code_TTT_1AQ_2AD

## Notes

Endoscopy E-Videos

https://eref.thieme.de/e-videos
*E-Videos* is an open access online section of the journal *Endoscopy* , reporting on interesting cases and new techniques in gastroenterological endoscopy. All papers include a high-quality video and are published with a Creative Commons CC-BY license. Endoscopy E-Videos qualify for HINARI discounts and waivers and eligibility is automatically checked during the submission process. We grant 100% waivers to articles whose corresponding authors are based in Group A countries and 50% waivers to those who are based in Group B countries as classified by Research4Life (see: https://www.research4life.org/access/eligibility/ ). This section has its own submission website at https://mc.manuscriptcentral.com/e-videos
